# The COVID-19 pandemic and family business performance

**DOI:** 10.1007/s11187-023-00766-2

**Published:** 2023-04-10

**Authors:** Ivan Miroshnychenko, Giorgio Vocalelli, Alfredo De Massis, Stefano Grassi, Francesco Ravazzolo

**Affiliations:** 1 IMD Business School, Ch. de Bellerive 23, P.O. Box 915, CH-1001 Lausanne, Switzerland; 2 Department of Economics, University of Verona, Via Cantarane 24, 37129 Verona, Italy; 3 Free University of Bozen-Bolzano, Piazza Università, 1, 39100 Bolzano, Italy; 4 Lancaster University Management School, Lancaster, UK; 5 Institute for Entrepreneurs and Institute of Family Business, Zhejiang University, Hangzhou, China; 6 Department of Economics and Finance, University of Rome Tor Vergata, Via Columbia 2, 00133 Rome, Italy; 7 Department of Data Science and Analytics, BI Norwegian Business School, Nydalsveien 37, 0484 Oslo, Norway; 8 Faculty of Economics and Management, Free University of Bozen-Bolzano, Piazza Università, 1, 39100 Bolzano, Italy

**Keywords:** Family firms, Financial performance, COVID-19, Pandemic, Resilience, G30, G32, G34

## Abstract

This study examines the impact of the COVID-19 pandemic on corporate financial performance using a unique, cross-country, and longitudinal sample of 3350 listed firms worldwide. We find that the financial performance of family firms has been significantly higher than that of nonfamily firms during the COVID-19 pandemic, accounting for pre-pandemic business conditions. This effect is pertinent to firms with strong family involvement in management or in both management and ownership. We also identify the role of firm-, industry-, and country-level contingencies for family business financial performance during the COVID-19 pandemic. This study offers a novel understanding of the financial resilience across different types of family business and sets an agenda for future research on the drivers of resilience of family firms to adverse events. It also provides important and novel evidence for policymakers, particularly for firms with different ownership and management structures.

## Abstract (plain language summary)

The COVID-19 pandemic spread at an unprecedented speed and scale that had not been seen since the Great Depression. But is there an organizational type that has been more financially resilient to the pandemic than others in the business landscape? We conducted a global study of 3350 publicly listed from 2018 to 2021 and found that family firms had substantially higher financial performance than nonfamily firms during the COVID-19 pandemic. Our main finding suggests that when governments develop and implement pandemic-related financial support programs for businesses, they need to take into account that the programs that are most suitable for family and nonfamily firms vary, due to the varying ability of both types of firms to be financially resilient in time of crisis. Investors must also pay attention to a firm’s ownership and management structure in evaluating its resilience—or otherwise—to future crises.

## Introduction

The COVID-19 pandemic directly affected many family firms around the world through a decline in sales and customer base (Belitski et al., 2022), distortion of their traditional logistic and distribution channels (Czakon et al., 2022), decreasing health and well-being of their workforce (Firfiray & Gomez-Mejia, 2021), blocking entire industries (Khlystova et al., 2022) and whole countries in times of national lockdowns (Calabrò et al., 2021). As family firms are known to survive tough economic times and prosper in the business landscape for very long periods (Bertrand & Schoar, 2006; Conz et al., 2020; Kansikas, 2015), they are a particularly interesting organizational setting to study a firm’s ability to respond to and recover from environmental shocks (Lengnick-Hall & Beck, 2016; O’Boyle et al., 2012). Unlike nonfamily firms, their behavior is anchored to a long-term orientation (James, 1999; Le Breton-Miller & Miller, 2006) rooted in their intention to transfer the business across generations (Zellweger et al., 2012).

The long-term orientation of family firms encompasses futurity, continuity, and perseverance (Lumpkin & Brigham, 2011; Lumpkin et al., 2010). These three distinct features lead family firms to prioritize long-term business goals and be better equipped to face ordinary business adversities (Fang et al., 2018; Lumpkin et al., 2010). As such, several scholars argue that family firms are more resilient than their nonfamily counterparts in normal economic times (Chrisman et al., 2011; Conz et al., 2020), but the question of whether a family firm’s superior ability to muddle through adversities holds when facing an unprecedented global health crisis remains largely unexplored, except for a few single-country studies (Amore et al., 2022; Carletti et al., 2020).

This study, therefore, examines the impact of the COVID-19 pandemic on the financial performance of family and nonfamily firms worldwide. To answer the question of their resilience in the pandemic, we use a longitudinal sample of 3350 listed firms in 33 countries and 10 industrial sectors over the period from 11 September 2018 to 9 September 2021.

Our findings show that family firms exhibited higher financial performance than nonfamily firms during the pandemic period, accounting for various firm-, industry-, and country-level differences. This effect is pertinent to firms with strong family involvement in management or both management and ownership. We also explored the role of firm-, industry-, and country-level contingencies of family business performance during the pandemic, accounting for pre-pandemic business conditions. Specifically, we found that the superior financial performance during the pandemic was largely driven by smaller and younger family firms from non-industrial sectors with low concentration in non-Anglo-Saxon countries. With a rich body of evidence from around the world, our results demonstrate the significant positive effect of family involvement in the business on financial performance during the pandemic, especially when the controlling family is actively involved in management or in both ownership and management; however, this superior ability varies substantially across different types of family firms, different industries, and different countries.

Our study contributes to the literature in at least three important ways. Prior family business studies have shown that family firms have better coped with financial crises in the USA (Zhou et al., 2017) and Italy (Minichilli et al., 2016). To our best knowledge, this is one of the first longitudinal studies to uncover the superior ability of the most ubiquitous form of entrepreneurial organizations—family firms—to resist the financial hardships caused by the COVID-19 pandemic, showing the importance of a concentrated ownership structure and management with long-term goals under environmental shocks on a global basis. We also compare the financial performance of different types of family firms in response to the COVID-19 pandemic and identify the most resilient types among family firms in managing the effects of the pandemic. Accordingly, we address the research calls of Kraus et al. (2020) and Calabrò et al. (2021) for large-scale empirical studies to capture the economic effects of the COVID-19 pandemic for family firms. In addition, we provide a global view of the financial responses of family firms to the COVID-19 pandemic extending growing single-country research in this area (Amore et al., 2022; Carletti et al., 2020).

Our study also contributes to the growing literature on the crisis management in the context of family firms (Conz et al., 2023; De Massis & Rondi, 2020; Czakon et al., 2022; Firfiray & Gomez-Mejia, 2021; Smith et al., 2023). We advance this literature by identifying the specific organizational ownership and management structure that exhibited financial resilience under adverse environmental conditions and, thereby, addressing the research call of Linnenluecke (2017). We further shed light on the roles of firm age and size, industry type and its concertation, as well as the geographical location of family firms for coping financially with the COVID-19 pandemic, thereby providing a detailed understanding of the specific types of moderating conditions ensuring a financial resilience of family firms during the event of crisis.

Finally, this study contributes to the regulatory, business, and academic debate on policy responses to the COVID-19 pandemic (Kurowski et al., 2020; OECD, 2020). By identifying the impact of family involvement on financial performance and the most resilient types of family firms during the pandemic, we provide important and novel evidence for policymakers, encouraging the implementation of fiscal and economic policies for COVID-19 recovery worldwide, particularly for firms with different ownership and management structures. Family-owned firms and nonfamily firms are likely to require more financial support because they demonstrated less resilience than family-managed and family-owned and managed firms during the pandemic. Moreover, it underscores the importance for policymakers to consider the role of the dominant owners and management structure to fully understand an organization’s ability to cope with adverse environmental shocks.

## Theory and hypotheses development

### “Shock-absorber” hypothesis

Unlike nonfamily firms that focus primarily on short- and medium-term goals when making strategic decisions, family firms concentrate on the business’s long-term success (Le Breton-Miller & Miller, 2006; Memili et al., 2018). This long-term orientation of family businesses encompasses three core elements: futurity, continuity, and perseverance (Brigham et al., 2014; Lumpkin & Brigham, 2011; Lumpkin et al., 2010).

Futurity reflects the ability to forecast and anticipate the consequences of business decisions in the long term. The family’s transgenerational perspective of the business is a clear example of futurity, as it entails the controlling family’s evident desire to pass the business on to the next generation (De Massis et al., 2012; James, 1999), thereby assuring the family’s long-term involvement in the business. In fact, several studies show that family owners consider long-term planning as pivotal for their business (Chua et al., 2003; Zellweger et al., 2012) and invest generously in the firm for the benefit of their descendants (Kappes & Schmid, 2013; Zellweger et al., 2013). On the contrary, the economic goals contained in the contracts of nonfamily firms’ managers are typically designed to align the interests of managers with those of investors (Barkema & Gomez-Mejia, 1998; Gomez-Mejia & Wiseman, 1997), putting enormous pressure on nonfamily firms to achieve short-term and medium-term economic goals to the exclusion of long-term objectives. Another example of futurity in family business is a strong family vision, “a notion of a better future for the family” (Chua et al., 1999, p. 24), that the controlling family develops and renews over time. Firm owners envision that the firm will continue to operate in the future, achieving the family’s desired growth rate and financial outcomes. The family firm’s vision reinforces the transgenerational perspective of creating long-term value for society and the family. In nonfamily firms, financial market pressures to achieve and exceed economic targets and eschew actions that fail to support those targets are likely to encourage a laser-like focus on economic success. Indeed, the reputation of managers of nonfamily firms is more likely to rest on the organization’s economic success than on how the organization envisions and manages stakeholder relations in pursuit of that success.

The continuity of the family business reflects the firm’s preservation and durability over time (Lumpkin & Brigham, 2011). The interplay between family firm resources and capabilities and their embeddedness in social, economic, and productive structures within their territories (Baù et al., 2019; Guenther et al., 2022) can create unique organizational continuity, a source of competitive advantage. This interplay can further help family firms build a distinct and durable family-based brand, including family members and the “extended family” of external stakeholders, such as workers, financers, suppliers, and customers (Sorenson et al., 2009). Therefore, these firms continuously invest in the future of the business, strengthening relations with their workers via generous training programs and more employee-oriented policies (Kang & Kim, 2020), and establishing durable relational links with external stakeholders (Orth & Green, 2009). In addition, family firms spend enormous financial resources on business renewal with the help of new product offerings and novel market extensions (De Massis et al., 2015; Miller et al., 2007a, 2007b). In contrast, managers of nonfamily firms are less likely than family owners and family managers to care that much about the continuity of the firm due to their typically shorter tenure. Nonfamily firms are unlikely to foster unique organizational continuity; instead, they are likely to engage in transactional relationships in which the short-term value of the economic exchange between employees, external stakeholders, and the firm determines whether to maintain or end these relationships.

Family business perseverance derives from their extraordinary regional embeddedness (Belitski & Rejeb, 2022; Guenther et al., 2022). This embeddedness helps them develop and maintain unique social capital—in which the competitive advantage of many family firms is rooted—by creating and maintaining extraordinary employee—(Azoury et al., 2013), and socially friendly policies and business practices (Dyer & Whetten, 2006). In turn, their greater commitment allows family firms to reach new market segments and higher profit margins, given that consumers are willing to pay extra for responsible products and services (Lanzini et al., 2016), especially from family firms perceived as trustworthy and quality-driven. Empirically, family business perseverance, on average, translates into higher firm-specific profitability over time and superior market value compared to nonfamily firms, except for the cluster of descendant-led family firms that are known to underperform (Pérez-González, 2006; Pindado & Requejo, 2015; Villalonga & Amit, 2006). On the other hand, nonfamily firms are more flexible in adjusting employment levels and socially friendly initiatives up and down as they see fit. Nonfamily firms have higher cash holdings than family firms (Moolchandani & Kar, 2022). This allows them to view the firm’s personnel and socially friendly initiatives as variable expenses that can be ratcheted up or down as economic conditions warrant. Thus, responding to the fiscal obligation to external shareholders combined with their ability to adjust the firm’s personnel and socially friendly initiatives, especially in the context where long-term goals are less important, managers of nonfamily firms have strong incentives to shift economic risk onto non-shareholder stakeholders such as employees and local community, thus using them as a buffer against changing economic and competitive circumstances that could negatively impact profits during the pandemic. Thus, business perseverance will be less pronounced in nonfamily firms compared to their family counterparts.

To sum up, we argue that family firms, thanks to their long-term orientation, are better equipped to respond to and recover from the shocks caused by the COVID-19 pandemic than nonfamily firms.

- Hypothesis 1 (H1): Family firms exhibit higher financial performance during the pandemic than nonfamily firms.

### Family involvement in ownership and/or management

Family involvement in a firm can take various forms (Chua et al., 1999; De Massis et al., 2012; Miller & Le Breton-Miller, 2005). Family involvement in ownership allows the controlling family to influence the firm’s strategic decisions and operations (Barontini & Caprio, 2006; Claessens et al., 2002; Faccio & Lang, 2002; Singal & Singal, 2011). The presence of family members in the firm’s top management allows the controlling family to exert an even stronger influence on the firm’s strategic decisions and operations than the sole ownership (Bozzi, Barontini, & Miroshnychenko, 2017; Kotlar & De Massis, 2013; Sanchez‐Bueno, Muñoz‐Bullón, & Galan, 2019). Furthermore, family involvement in both ownership and management (Anderson & Reeb, 2003a, 2003b; Muñoz-Bullon, Sanchez-Bueno, & Suárez-González, 2018; Yu, Lumpkin, Sorenson, & Brigham, 2012) allows the controlling family to have absolute control over the firm (Zellweger et al., 2012). In fact, various studies show that family goals and vision (Chua et al., 1999; De Massis et al., 2018a, 2018b) are highly correlated with the extent of family involvement in ownership and management (Chrisman et al., 2012; Chrisman & Patel, 2012).

In contrast to family-managed or family-owned and managed firms, family-owned firms lack the possibility to actively influence the firm’s strategic decisions and operations through family managers, thus making the prioritization of long-term goals less pronounced in this type of family business. A long-term orientation induces family members in managerial positions to invest in the firm’s resources to deliver more valuable output (Miller et al., 2008, 2007a, 2007b). Family managers identify with the firm and benefit from psychological ownership that pushes them to search for better solutions to business issues (Rau et al., 2019), particularly in rough economic times (Zhou et al., 2017). In other words, family managers in firms with family involvement in management or both management and ownership are likely to positively influence strategic decisions aimed at superior financial returns, particularly during the COVID-19 pandemic. Increased commitment of family managers to the business also means greater managerial attention and control over the use of resources (i.e., better resource orchestration). In turn, attention allocation (of family managers), alongside better resource orchestration, helps them to effectively manage knowledge and business opportunities, renewing the organizational identity, and coping with uncertainty, which is especially useful for muddling through adversities (Conz et al., 2020; De Massis & Rondi, 2020; Lengnick-Hall & Beck, 2016). Expecting lengthy tenures, family managers are also less apt to make fast decisions to impress the board compared to their shorter-term peers (Le Breton-Miller & Miller, 2015). In fact, strategic decisions related to long-term projects and efficient resource allocation to increase “returns over a prospectively lengthy career” are often preferred (James, 1999). Therefore, firms with family involvement in management or both management and ownership, thanks to the presence of long-term-oriented family managers, will be more equipped than others to respond to and recover from the COVID-19 pandemic.

- Hypothesis 2 (H2): The positive effect of family involvement during the pandemic will be substantially higher for family-managed firms and family-owned and managed firms, as compared to nonfamily firms.

### Founders and descendants

A vast amount of literature documents the substantial differences in the corporate financial performance of founder-led vs. descendant-led family firms (Barontini & Caprio, 2006; Miller et al., 2007a, 2007b; Miller et al., 2007a; Villalonga & Amit, 2006).

Founder-led family firms have, on average, higher market valuations and better financial performance than descendant-led family firms (Barontini & Caprio, 2006; Villalonga & Amit, 2006). The higher financial returns of founder-led family firms allow retaining a strong position in the marketplace and weathering environmental shocks. In contrast, descendant-led family firms may generate financial returns by simply maintaining the core business without pushing the firm’s performance boundaries. Furthermore, founder-led family firms can excel in performing above the norm not only due to the need to develop the business while maintaining family control, but also as a result of the presence of the founder within the firm (Chirico et al., 2011; Miller et al., 2007a, 2007b; Pryor et al., 2019). Founders create a company vision, inspire employees, develop products and services based on their vision, and perform management tasks and duties essential to growing the business (Wasserman, 2003). They are entrepreneurs with the necessary level of alertness, leadership, temperament, and profound knowledge of the core business activities needed to explore opportunities. Even when the pressure on short-term results is high, firms actively managed by their founders invest heavily (Kappes & Schmid, 2013; Veider & Kallmuenzer, 2016; Wasserman, 2003), particularly in times of crisis when a firm’s continuous investments are the driving force of its survival and prosperity in the long term.

However, negative forces are at work that can limit the firm’s potential to withstand environmental shocks when descendants run publicly listed firms. First, heirs’ control over a firm does not guarantee inherited talent or business skills, but rather signals simple kinship (Pérez-González, 2006). Second, descendants tend to adopt poor monitoring, target, and incentive management practices (Bloom et al., 2011; Tsoutsoura, 2021) to the detriment of financial performance as a result of the less motivated and less productive labor force. Third, some studies suggest that financial performance and firm value are destroyed when descendants are in charge of the firm (Cucculelli & Micucci, 2008; Miroshnychenko et al., 2021; Villalonga & Amit, 2006), and their prospects of survival might be hampered by the lesser availability of internal financing and the lower ability to raise external financing. This issue is particularly harmful for later-generational family firms when descendants exert sufficient control to maintain their position within a firm despite their incompetence (Claessens et al., 2002).

Given the aforementioned negative attributes typically attributed to the presence of heirs at the helm of the firm, we expect that descendants-led firms will be able to worsen their response to and recover from the financial shocks caused by the pandemic, compared to founder-led firms. Thus, our last hypothesis can be stated as the following:

- Hypothesis 3 (H3): The positive effect of family involvement during the pandemic will be substantially higher for founder-led family firms, as compared to descendants-led family firms.

## Data

### Sources

The starting point of our data collection was the NRG Metrics’ Family Firms dataset, manually developed by an expert team. NRG Metrics uses publicly available documents (annual reports, firm presentations, SEC filings, and press releases) as data sources. All levels of data entry are cross-checked for inconsistencies and errors using sophisticated software programs (NRG Metrics, 2021). NRG has been validated in both management and finance literature (Cho et al., 2019; Dal Maso et al., 2020; Delis et al., 2020). Then, we collected financial and accounting data from Thomson Reuters Eikon. The COVID-19 data derives from the COVID-19 Data Repository of the Center for Systems Science and Engineering at Johns Hopkins University. We eliminated firms with missing financial, accounting, or pandemic data from the sample following common practice in the field. As a result, our final dataset covers 3350 listed firms in 33 countries and 10 industrial sectors from 11 September 2018 to 9 September 2021.

**Fig. 1:**
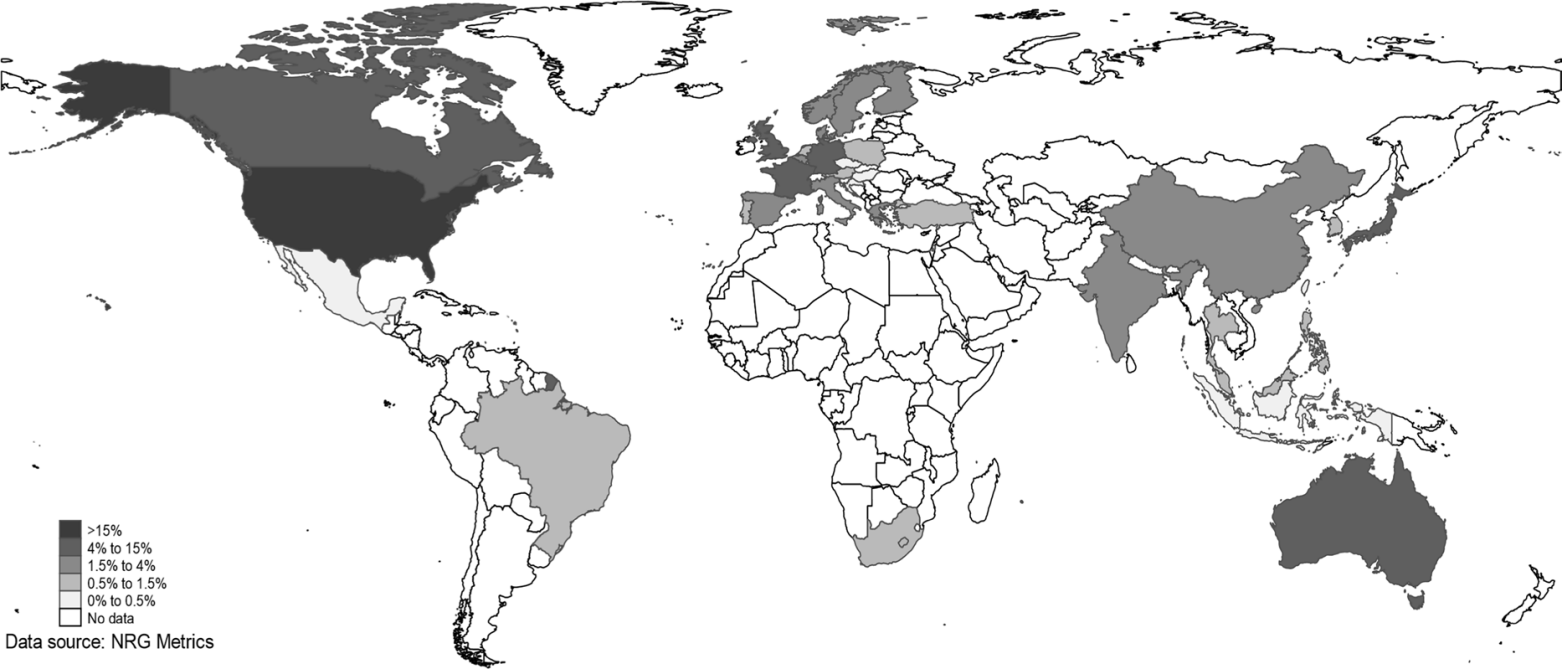
Distribution of the sample by country

**Fig. 2:**
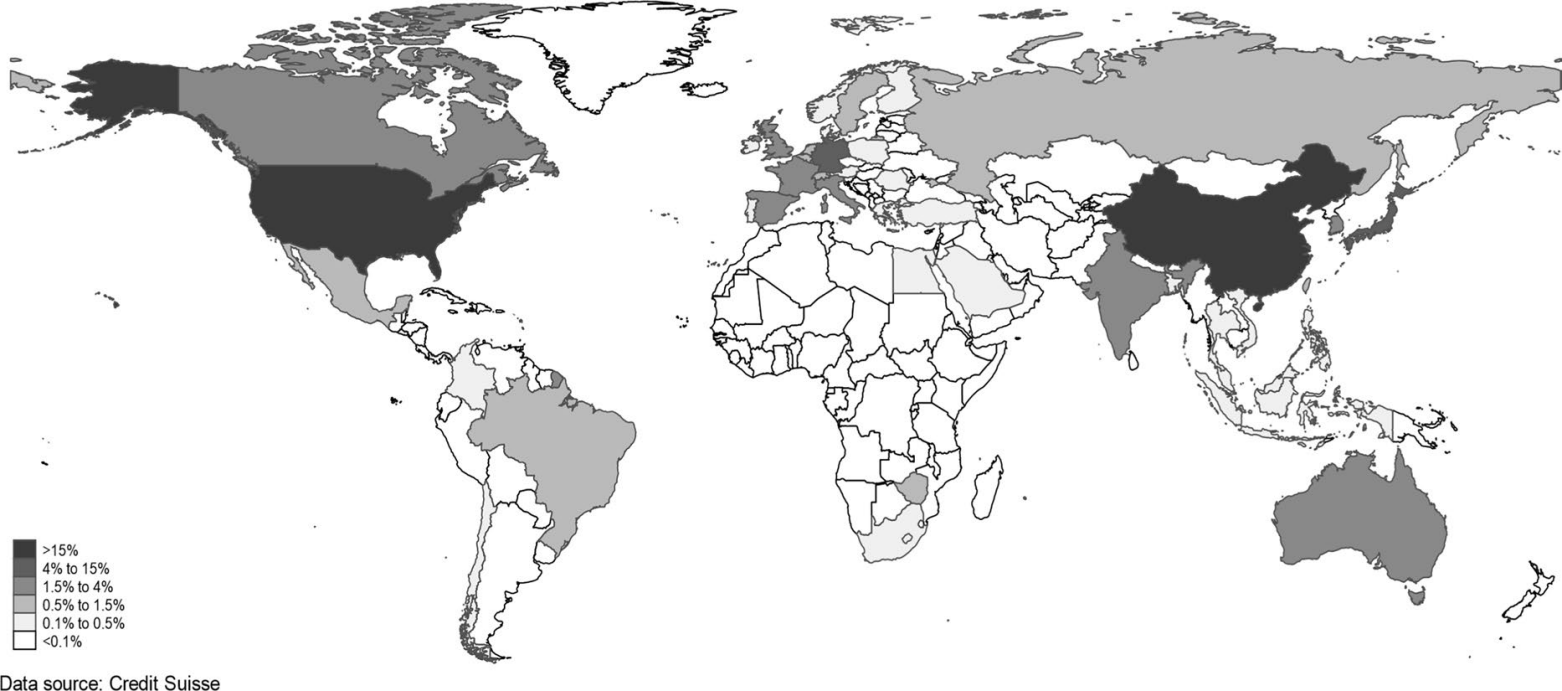
Distribution of the share of total wealth by country

Figure 1 shows the composition of our sample by country.^1^ Table 1 further shows the distribution of family and nonfamily firms across countries. The largest share of publicly traded firms is from Anglo-Saxon (47%), European (38%), and Asian countries (12%).^2^^2^ The rest of the sample is broadly distributed among the other countries. Our sample closely resembles the global wealth distribution shown in Fig. 2 (Credit Suisse, 2019). Industrial (around 27%), consumer services (15%), consumer goods (14%), basic materials (10%), and technology (9%) constitute the largest share of firms in our sample. The remainder of the sample is broadly distributed among other industries.

**Table 1:** Distribution of family and nonfamily firms by geographical region (%)

	Nonfamily firms	Family firms	Total
Anglo-Saxon countries	52.43	34.38	47.12
European countries	32.05	51.16	37.68
Asian countries	12.23	10.68	11.77
Others	3.29	3.79	3.44
Total	100.00	100.00	100.00

Anglo-Saxon countries include Australia, Canada, the UK, and the USA. European countries include Austria, Belgium, Denmark, Finland, France, Germany, Greece, Hungary, Italy, the Netherlands, Norway, Poland, Portugal, Spain, and Sweden. Asian countries include China, Indonesia, Japan, Malaysia, Philippines, Singapore, South Korea, Taiwan, and Thailand. Others include Brazil, Israel, Mexico, South Africa, and Turkey

### Variables

#### Financial performance

We adopt daily abnormal stock returns as the proxy of financial performance. Using closing prices, it is estimated as a variance between the actual return for a stock and the return based on market expectations for firm *i* from country *c* between time *t – 1* and *t*.

#### COVID-19 growth

We calculate the COVID-19 growth variable as the following: (confirmed COVID-19 cases for country *c* at time *t –* confirmed COVID-19 cases for country *c* at time *t–1*) / confirmed COVID-19 cases for country *c* at time *t–1*)*.* Given that the confirmed COVID-19 cases are registered and reported at the end of the day, the reactions of financial markets follow the next day. To incorporate this time lag between COVID-19 growth and stock price movements, we lag the COVID-19 growth variable.

#### Family firms

Family involvement in the firm’s ownership and/or management is commonly used to operationalize family business (Chua et al., 1999; Kotlar et al., 2014, 2018; Matzler et al., 2015). Accordingly, family firms are those firms where the founding family has equity ownership more than 5% and/or family members serving on the board of directors (Chrisman & Patel, 2012; Patel & Chrisman, 2014). Following Anderson & Reeb (2003a, 2003b), this dummy variable equals 1 if the founder, descendant, or family member is a director or large shareholder, and 0 otherwise. We also distinguish between different types of family firms, capturing family-owned firms (dummy variable that equals 1 for a firm with family equity ownership more than 5%, 0 otherwise), family-managed firms (dummy variable that equals 1 for a firm with family members serving on the board of directors, 0 otherwise), and family-owned and managed firms (dummy variable that equals 1 for a firm with family equity ownership more than 5% and family members serving on the board of directors, 0 otherwise). In addition, the variables founder CEO (dummy variable that equals 1 for a firm with the founder serving as CEO, 0 otherwise) and descendant CEO (dummy variable equals 1 for a firm with descendant serving in the role of CEO, 0 otherwise) capture the generational involvement of the family in the business.

### Control variables

We use a vector of control variables, common in the literature, to account for firm-, industry-, and country-level differences in our sample that might affect firm performance (De Massis et al., 2012, 2020; Soluk et al., 2021). Given the evidence that financial indebtedness adversely affects firm profitability, we construct a proxy of *financial leverage* (ratio of total debt to total assets) (Opler & Titman, 1994). We also control for the firm’s ability to generate funds internally by including a proxy of *cash flows* (ratio of net income and non-cash charges to total assets) (Barontini & Caprio, 2006). *Firm age* (the natural logarithm of years for which firm exists) and *firm size* (the natural logarithm of number of employees) are included as older and larger firms accumulate learning and resources that may enhance their performance (Beck et al., 2005). Financial leverage, cash flows, and firm size refer to 2018 to avoid simultaneity problem. Therefore, these variables are treated as time-invariant in our explanatory model.

Moreover, for each country, we create a dummy variable (*policy*) taking value equal to 1 if corporate asset purchases programs were introduced by policy authorities in the months following the official declaration of pandemic by World Health Organization (WHO), and equal to zero otherwise (Source of data: COVID-19 Financial Response Tracker at Yale University).

We further control for systematic differences in financial performance across different firms, industrial sectors, and countries by including *firm-level*, *industry-level* (1-digit ICB codes), and *country-level fixed effects* in our model (De Massis et al., 2017, 2020; Dess et al., 1990). It is worth noting that we do not include time-fixed effects due to alleviating most of the variation in our COVID-19 growth variable.

## Method

To examine the average effect of the COVID-19 pandemic on the financial performance of family and nonfamily firms, we adopt the panel data methodology clustering standard errors at the firm level, as suggested by Petersen (2009). This methodology allows us to identify the time-varying relationship between our dependent and independent variables, accounting for firm-, industry-, and country-level differences in our sample. First, we estimate our explanatory model using the random-effects (RE) estimator, allowing us to capture the effect of time-invariant variables in the model. Then, we further apply the fixed-effects (FE) estimator, allowing us to control for firm-level unobservable heterogeneity, which is an important source of the endogeneity problem in the family business research (Evert et al., 2016). Given that the proxies of family involvement, financial leverage, cash flows, and firm size are time-invariant, they are automatically dropped in the FE regressions; however, we can still estimate the interaction terms between the various proxies of family involvement and the COVID-19 growth variables. Please note that mixed-frequency models are not suitable for our research settings due to extremely low variation in control variables across different business quarters.

### Descriptive statistics and correlations

Tables 2 and 3 report the descriptive statistics and pairwise correlations. Family firms represent around 29% of our sample, in line with prior studies on family-controlled publicly traded firms (Anderson & Reeb, 2003a, 2003b; Villalonga & Amit, 2006), of which 10% are family-owned, 7% are family-managed, and 12% have family involvement in both ownership and management. Founder-led family firms constitute around 2% and descendant-led family firms 5% of the sample.

**Table 2:** Descriptive statistics

	Mean	S.D	Min	Max
COVID-19 growth	0.025	0.176	− 0.318	17.000
Financial performance	− 0.003	2.518	− 75.016	231.477
Family firms	0.295	0.456	0.000	1.000
Family-owned firms	0.100	0.300	0.000	1.000
Family-managed firms	0.071	0.257	0.000	1.000
Family-managed-owned firms	0.124	0.329	0.000	1.000
Descendant CEO	0.053	0.224	0.000	1.000
Founder CEO	0.018	0.133	0.000	1.000
Financial leverage	0.254	0.237	0.000	6.571
Cash flows	0.087	0.124	− 1.460	0.681
Firm age	3.867	0.779	0.000	6.225
Firm size	8.440	1.987	0.693	14.604
Policy	0.426	0.494	0.000	1.000

**Table 3:** Correlations

	1	2	3	4	5	6	7	8	9	10	11	12	13
1 COVID-19 growth	1												
2 Financial performance	0.00	1											
3 Family firms	0.00	0.00	1										
4 Family-owned firms			0.52	1									
5 Family-managed firms		0.00	0.43	− 0.09	1								
6 Family-owned-managed firms		0.00	0.58	− 0.13	− 0.10	1							
7 Descendant CEO		0.00	0.37	− 0.08	0.86	− 0.09	1						
8 Founder CEO			0.21	− 0.05	0.49	− 0.05	− 0.03	1					
9 Financial leverage	0.00	0.00	− 0.04	0.00	− 0.02	− 0.04	− 0.02	0.00	1				
10 Cash flows	0.00	0.01	− 0.05	0.01	− 0.03	− 0.06	− 0.02	− 0.04	− 0.13	1			
11 Firm size		0.00	− 0.09	0.01	− 0.03	− 0.11	0.02	− 0.10	0.11	0.23	1		
12 Firm age	0.00		− 0.11	− 0.02	− 0.09	− 0.07	− 0.04	− 0.10	0.02	0.11	0.34	1	
13 Policy	− 0.05	0.00	− 0.01	0.00	− 0.02	0.01	− 0.03	0.00	− 0.01	0.01	0.01	0.0	1

Displayed correlation coefficients are statistically significant at the 5% level

The variance inflation factors (VIFs) never exceed 2, suggesting that multicollinearity is not a concern. The results of the unit root test of Levine et al. (2002), applied to COVID-19 growth and financial performance variables, are reported in Table 4. We reject the null hypothesis for both variables, suggesting that these data points are stationary.

**Table 4:** Unit root test

Unit-root test	*t*-statistic	*p*-value
Financial performance	− 1200.00	0.00
COVID-19 growth	− 79.22	0.00

Levin-Lin-Chu test H_0_: Panels contain unit root

## Results

### Main results

Table 5 presents the results of the RE and FE regressions of the relationship between family involvement in the firm and firm performance during the COVID-19 pandemic. Models 1–2 assess the average impact of the COVID-19 pandemic on the financial performance of listed firms worldwide. Models 3–8 further reveal the average impact of the COVID-19 pandemic on the financial performance of different types of family firms vs. nonfamily firms worldwide.

**Table 5:** Main results

Estimator:	RE	FE	RE	FE	RE	FE	RE	FE
Dependent variable: financial performance	Model (1)	Model (2)	Model (3)	Model (4)	Model (5)	Model (6)	Model (7)	Model (8)
Financial leverage	− 0.0099		− 0.0095		− 0.0095		− 0.0095	
	(0.243)		(0.268)		(0.268)		(0.269)	
Cash flows	0.1345		0.1348		0.1347		0.1347	
	(0.000)		(0.000)		(0.000)		(0.000)	
Firm size	0.0015		0.0016		0.0016		0.0016	
	(0.096)		(0.087)		(0.086)		(0.086)	
Firm age	− 0.0022	0.1795	− 0.0020	0.1808	− 0.0021	0.1805	− 0.0021	0.1805
	(0.292)	(0.000)	(0.338)	(0.000)	(0.312)	(0.000)	(0.315)	(0.000)
Policy	0.0159	0.0074	0.0158	0.0073	0.0158	0.0073	0.0158	0.0073
	(0.000)	(0.014)	(0.000)	(0.015)	(0.000)	(0.015)	(0.000)	(0.015)
COVID-19 growth	− 0.0363	− 0.0272	− 0.0658	− 0.0583	− 0.0678	-0.0582	− 0.0678	− 0.0582
	(0.003)	(0.021)	(0.000)	(0.000)	(0.000)	(0.000)	(0.000)	(0.000)
Family firms			0.0027					
			(0.446)					
Family firms x COVID-19 growth			0.0880	0.0913				
			(0.000)	(0.000)				
Family-owned firms					0.0040		0.0040	
					(0.446)		(0.445)	
Family-managed firms					− 0.0009			
					(0.889)			
Family-managed-owned firms					0.0030		0.0030	
					(0.559)		(0.557)	
Family-owned firms x COVID-19 growth					0.0512	0.0340	0.0512	0.0340
					(0.154)	(0.331)	(0.154)	(0.331)
Family-managed firms x COVID-19 growth					0.0942	0.0856		
					(0.024)	(0.033)		
Family-managed-owned firms x COVID-19 growth					0.1319	0.1433	0.1319	0.1433
					(0.000)	(0.000)	(0.000)	(0.000)
Descendant CEO							− 0.0014	
							(0.854)	
Founder CEO							0.0004	
							(0.976)	
Descendant CEO x COVID-19 growth							0.0989	0.0872
							(0.041)	(0.066)
Founder CEO x COVID-19 growth							0.0821	0.0819
							(0.250)	(0.230)
Firm FE	No	Yes	No	Yes	No	Yes	No	Yes
Industry FE	Yes	Yes	Yes	Yes	Yes	Yes	Yes	Yes
Country FE	Yes	Yes	Yes	Yes	Yes	Yes	Yes	Yes
Constant	− 0.0171	− 0.6991	− 0.0183	− 0.7040	− 0.0180	− 0.7030	− 0.0181	− 0.7030
	(0.181)	(0.000)	(0.152)	(0.000)	(0.160)	(0.000)	(0.159)	(0.000)
Observations	2,350,117	2,597,534	2,350,117	2,597,534	2,350,117	2,597,534	2,350,117	2,597,534

Notes: This table presents the coefficients and *p*-values (in parentheses) using the RE and FE regressions with robust standard errors clustered at the firm level

**Fig. 3:**
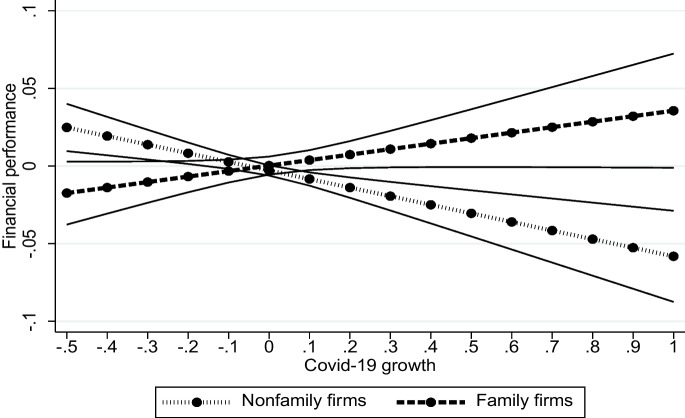
Marginal effects. Notes: This figure shows the average marginal effects of COVID-19 growth on the financial performance of family and nonfamily firms. The continuous lines are the 95% confidence bands

H1 postulates that family firms will exhibit higher financial performance during the pandemic period than nonfamily firms. The coefficient of the *COVID-19 growth* variable is negative and statistically significant (Model 3: *β* =  − 0.0658; *ρ* < *0.01;* Model 4: *β* =  − 0.0583; *ρ* < *0.01)*, suggesting that the pandemic, on average, has had a negative impact on the financial performance of nonfamily firms. This result confirms and expands the conclusions of the single-country study of Al-Awadhi et al. (2020). The interaction term between the *family firms* and *COVID-19 growth* variables is positive and highly statistically significant (Model 3: *β* = 0.0880; *ρ* < *0.01;* Model 4: *β* = 0.0913;* ρ* < *0.01)*. We further calculated the average marginal effects of the financial performance of family and nonfamily firms in Fig. 3 to interpret this interaction and verify its significance across different levels of COVID-19 growth. This figure shows the predicted financial performance as a function of a reduction in COVID-19 cases (up to 50%) and an increase in COVID-19 cases (up to 100%). As we can see, family business performance increases as the level of COVID-19 cases increases, while the financial performance of nonfamily counterparts is negatively affected. Moreover, this performance difference is statistically significant at a 95% confidence level, confirming H1. Moreover, when COVID-19 cases double, e.g., in the first phases of the COVID-19 pandemic, nonfamily firms face more than a 5 basis point (bp) of expected losses, while family firms experience almost a 5 bp expected gains, leading to a difference of 10 bp in just one day.

H2 predicts that the positive effect of family involvement during the pandemic will be substantially higher for family-managed firms and family-owned and managed firms, as compared to nonfamily firms. In Models 5–6, we consider different types of family involvement on firm performance and find that it is mainly family involvement in management (Model 5: *β* = 0.0942; *ρ* < *0.05*; Model 6: *β* = 0.0856; *ρ* < *0.05*) or both ownership and management (Model 5: *β* = 0.1319; *ρ* < *0.01*; Model 6: *β* = 0.1433; *ρ* < *0.01*) that brought value to financial performance during the pandemic. It appears that family-managed firms are able to achieve better financial performance not only in normal times (Miller et al., 2007a, 2007b), but also in times of environmental shocks. In addition, family-owned and managed firms are also better equipped to respond to and recover from the shocks caused by the COVID-19 pandemic. In contrast, the positive moderating effect vanishes when the controlling family is involved only in the firm’s ownership and becomes indistinguishable from nonfamily business performance. Hence, our H2 is confirmed.

H3 postulates that the positive effect of family involvement will be substantially higher for founder-led family firms, as compared to descendant-led family firms. Looking at Models 7–8, the interaction term between *founder CEO* and *COVID-19 growth* is not statistically different from zero, while the interaction term between *descendant CEO* and *COVID-19 growth* is positive and statistically significant (Model 7: *β* = 0.0989; *ρ* < 0.05*;* Model 8: *β* = 0.0872; *ρ* < *0.10*). This result provides some evidence indicating that descendant-led family firms had higher financial performance during the pandemic, as compared to nonfamily firms. However, the difference in the positive family business effect between descendant-led and founder-led family firms is not statistically significant, implying that H3 is rejected.

The supposition behind these results is that family involvement can bring value to the firm in adverse economic times, but this effect depends on the type of family involvement in the firm. Firms with strong family involvement either in management or in both management and ownership on average demonstrated better financial performance during the COVID-19 pandemic than nonfamily firms. However, the financial performance of descendant-led family firms during the pandemic did not substantially differ from that of founder-led firms.

### Robustness check

To verify the sensitivity of our findings, we re-estimated our explanatory model using alternative variable definitions of family firms (a dummy variable that equals to 1 for a firm where the founding family has equity ownership more than 10% and/or family members serving on the board of directors, 0 otherwise), family-owned firms (a dummy variable that equals to 1 for a firm with family equity ownership more than 10%, 0 otherwise), and family-owned and managed firms (dummy variables that equals to 1 for a firm with family equity ownership more than 10% and family members serving on the board of directors). As shown in Table 6, our main findings remain unchanged.

**Table 6:** Robustness check

Dependent variable: financial performance	Model (1)	Model (2)	Model (3)	Model (4)
COVID-19 growth	− 0.0272	− 0.0586	− 0.0599	− 0.0599
	(0.021)	(0.000)	(0.000)	(0.000)
Firm age	0.1795	0.1727	0.1726	0.1726
	(0.000)	(0.000)	(0.000)	(0.000)
Policy	0.0074	0.0069	0.0069	0.0069
	(0.014)	(0.022)	(0.022)	(0.022)
Family firms x COVID-19 growth		0.0914		
		(0.000)		
Family-owned firms x COVID-19 growth			0.0451	0.0451
			(0.200)	(0.200)
Family-managed firms x COVID-19 growth			0.0871	
			(0.031)	
Family-managed-owned firms x COVID-19 growth			0.1449	0.1449
			(0.000)	(0.000)
Descendant CEO x COVID-19 growth				0.0885
				(0.062)
Founder CEO x COVID-19 growth				0.0836
				(0.221)
Firm FE Industry FE Country FE	Yes Yes Yes	Yes Yes Yes	Yes Yes Yes	Yes Yes Yes
Constant	− 0.6991	− 0.6991	− 0.7040	− 0.7030
	(0.000)	(0.000)	(0.000)	(0.000)
Observations	2,597,534	2,597,534	2,597,534	2,597,534

Notes: This table presents the coefficients and *p*-values (in parentheses) using the FE regressions with robust standard errors clustered at the firm level. Family firms are those firms where the founding family has equity ownership more than 10% and/or family members serving on the board of directors. Family-owned firms are those firms with family equity ownership more than 10%. Family-managed firms are those firms with family members serving on the board of directors. Family-owned and managed firms are those firms with family equity ownership more than 10% and family members serving on the board of directors

### Post hoc analyses

Thus far, we have shown that, on average, the financial performance of family firms has been higher as that of nonfamily firms during the pandemic. We subsequently adopted the fact-based research approach following Hambrick (2007) and Miller (2007), particularly useful when scholars face an interesting phenomenon that no theory can fully or appropriately explain. The fact-based research approach allowed us to extract patterns from our empirical data that can yield insights on the role of firm-, industry-, and country- level contingencies of family business performance during the pandemic. We first analyze the moderating effects of firm size and firm age on the relationship between family influence and financial performance during the pandemic. Afterwards, we focus on the industry type and its concentration as the possible drivers behind the cross-sectional variation in the difference in financial performance between family and nonfamily firms operating in different industrial sectors. Finally, the differences between Anglo-Saxon countries, European countries, Asian countries, and other countries are explored to capture possible geographical variations.

Some research argues that firm size can play a moderating role in the relationship between family influence and the financial performance of family business (Wagner et al., 2015). Large family firms often have large markets to cover and, therefore, can generate higher profitability (De Massis et al., 2012). Moreover, they can take advantage of the economy of scale and reduce their average costs. At the same time, large family firms are more bureaucratic and less agile (Tipu, 2022), as compared to small family firms. Thus, in times of the COVID-19 pandemic, when both demand and supply have been largely distorted, large family firms, exposed to substantial financial losses coupled with lower ability to adjust their operations, might not have been able to outperform small family firms subject to lower financial losses and being able to swiftly adjust their operations. We tested this prediction on the subsample of large firms (with firm size above the sample median) in Table 7 and the subsample of small firms (with firm size below the sample median) in Table 8. Both large and small family-owned and managed firms exhibited superior financial performance during the pandemic, while the superior financial performance of family-owned firms and family-managed firms holds only in the subsample of small family firms. We also find that only small descendants-led family firms were able to outperform financially nonfamily firms during the pandemic. Thus, we find some empirical support to our prediction.

**Table 7:** Post-hoc analysis: variations across firms—Large firms

Dependent variable: financial performance	Model (1)	Model (2)	Model (3)	Model (4)
COVID-19 growth	− 0.0718	− 0.0934	− 0.0934	− 0.0934
	(0.0000)	(0.0000)	(0.0000)	(0.0000)
Firm age	0.1183	0.1192	0.1193	0.1191
	(0.0144)	(0.0137)	(0.0136)	(0.0137)
Policy	− 0.0020	− 0.0021	− 0.0021	− 0.0021
	(0.5954)	(0.5769)	(0.5764)	(0.5775)
Family firms x COVID-19 growth		0.0758		
		(0.0353)		
Family-owned firms x COVID-19 growth			0.0253	0.0253
			(0.6144)	(0.6144)
Family-managed firms x COVID-19 growth			0.0257	
			(0.6936)	
Family-managed-owned firms x COVID-19 growth			0.1550	0.1550
			(0.0037)	(0.0037)
Descendant CEO x COVID-19 growth				− 0.0040
				(0.9531)
Founder CEO x COVID-19 growth				0.2154
				(0.2087)
Firm FE Industry FE Country FE	Yes Yes Yes	Yes Yes Yes	Yes Yes Yes	Yes Yes Yes
Constant	− 0.4759	− 0.4798	− 0.4800	− 0.4792
	(0.0139)	(0.0132)	(0.0132)	(0.0133)
Observations	1,419,416	1,419,416	1,419,416	1,419,416

Notes: This table presents the coefficients and *p*-values (in parentheses) using the FE regressions with robust standard errors clustered at the firm level

**Table 8:** Post-hoc analysis: variations across firms—Small firms

Dependent variable: financial performance	Model (1)	Model (2)	Model (3)	Model (4)
COVID-19 growth	0.0116	− 0.0310	− 0.0310	− 0.0310
	(0.4772)	(0.1511)	(0.1512)	(0.1512)
Firm age	0.2185	0.2206	0.2205	0.2205
	(0.0006)	(0.0005)	(0.0005)	(0.0005)
Policy	0.0191	0.0189	0.0189	0.0189
	(0.0001)	(0.0001)	(0.0001)	(0.0001)
Family firms x COVID-19 growth		0.1180		
		(0.0004)		
Family-owned firms x COVID-19 growth			0.0867	0.0867
			(0.0744)	(0.0744)
Family-managed firms x COVID-19 growth			0.1083	
			(0.0350)	
Family-managed-owned firms x COVID-19 growth			0.1446	0.1446
			(0.0014)	(0.0014)
Descendant CEO x COVID-19 growth				0.1516
				(0.0118)
Founder CEO x COVID-19 growth				0.0374
				(0.6089)
Firm FE Industry FE Country FE	Yes Yes Yes	Yes Yes Yes	Yes Yes Yes	Yes Yes Yes
Constant	− 0.8151	− 0.8227	− 0.8223	− 0.8224
	(0.0005)	(0.0004)	(0.0004)	(0.0004)
Observations	1,177,335	1,177,335	1,177,335	1,177,335

Notes: This table presents the coefficients and *p*-values (in parentheses) using the FE regressions with robust standard errors clustered at the firm level

As family firms become older, they become more reluctant to change their day-to-day operational activities (Zahra et al., 2008) and, more importantly, become more rigid to the adoption of novel business practices and processes (Bloom et al., 2011). Thus, the ability of old family firms to swiftly adapt to adverse environmental conditions is likely to be weaker, as compared to young family firms. Therefore, it is likely that firm age can play an important moderating role in the relationship between family influence and financial performance during the pandemic when firms had to suddenly adjust their modus operandi to new business realities. To test this prediction, we have re-run all our estimations on the subsample of old firms (with firm age above the sample median) and the subsample of young firms (with firm age below the sample median) in Tables 9 and 10. Family-owned and managed firms exhibited superior financial performance during the pandemic regardless of their age, while only young family-owned firms and young family-managed firms were able to outperform nonfamily firms during the pandemic. In addition, we observe that only young descendants-led family firms were able to deliver the superior financial performance compared to nonfamily firms during the pandemic. Hence, we find some empirical evidence in favor of our prediction.

**Table 9:** Post-hoc analysis: variations across firms—Old firms

Dependent variable: financial performance	Model (1)	Model (2)	Model (3)	Model (4)
COVID-19 growth	− 0.0492	− 0.0642	− 0.0642	− 0.0642
	(0.0012)	(0.0005)	(0.0005)	(0.0005)
Firm age	0.8957	0.9015	0.9022	0.9023
	(0.0000)	(0.0000)	(0.0000)	(0.0000)
Policy	− 0.0030	− 0.0031	− 0.0031	− 0.0031
	(0.5360)	(0.5178)	(0.5146)	(0.5144)
Family firms x COVID-19 growth		0.0522		
		(0.1058)		
Family-owned firms x COVID-19 growth			0.0109	0.0109
			(0.8098)	(0.8098)
Family-managed firms x COVID-19 growth			0.0542	
			(0.3676)	
Family-managed-owned firms x COVID-19 growth			0.0916	0.0916
			(0.0525)	(0.0525)
Descendant CEO x COVID-19 growth				0.0524
				(0.4623)
Founder CEO x COVID-19 growth				0.0607
				(0.4985)
Firm FE Industry FE Country FE	Yes Yes Yes	Yes Yes Yes	Yes Yes Yes	Yes Yes Yes
Constant	− 4.0349	− 4.0608	− 4.0640	− 4.0643
	(0.0000)	(0.0000)	(0.0000)	(0.0000)
Observations	1,311,933	1,311,933	1,311,933	1,311,933

Notes: This table presents the coefficients and *p*-values (in parentheses) using the FE regressions with robust standard errors clustered at the firm level

**Table 10:** Post-hoc analysis: variations across firms—Young firms

Dependent variable: financial performance	Model (1)	Model (2)	Model (3)	Model (4)
COVID-19 growth	− 0.0065	− 0.0646	− 0.0646	− 0.0646
	(0.7232)	(0.0097)	(0.0097)	(0.0097)
Firm age	0.1632	0.1670	0.1671	0.1671
	(0.0002)	(0.0002)	(0.0002)	(0.0002)
Policy	0.0063	0.0059	0.0059	0.0059
	(0.2217)	(0.2554)	(0.2550)	(0.2548)
Family firms x COVID-19 growth		0.1558		
		(0.0000)		
Family-owned firms x COVID-19 growth			0.1099	0.1099
			(0.0484)	(0.0484)
Family-managed firms x COVID-19 growth			0.1216	
			(0.0285)	
Family-managed-owned firms x COVID-19 growth			0.2050	0.2050
			(0.0000)	(0.0000)
Descendant CEO x COVID-19 growth				0.1332
				(0.0330)
Founder CEO x COVID-19 growth				0.1016
				(0.2756)
Firm FE Industry FE Country FE	Yes Yes Yes	Yes Yes Yes	Yes Yes Yes	Yes Yes Yes
Constant	− 0.5275	− 0.5396	− 0.5399	− 0.5398
	(0.0002)	(0.0002)	(0.0002)	(0.0002)
Observations	1,285,601	1,285,601	1,285,601	1,285,601

Notes: This table presents the coefficients and *p*-values (in parentheses) using the FE regressions with robust standard errors clustered at the firm level

Following a recent study on family business sector-related determinants (De Massis et al., 2017; Khlystova et al., 2022), we classified all industries in our sample into industrial and non-industrial sectors. The main rationale behind this classification is that family firms operating in non-industrial sectors (i.e., hospitality sector) have experienced higher financial losses during the pandemic compared to family firms in industrial sectors that in most of cases continued conducting their daily operations. After re-estimating our main explanatory model using the two subsamples, we find that family-owned and managed firms have outperformed financially nonfamily firms in both industrial and non-industrial sectors (see Tables 11 and 12). However, family-managed firms were able to better respond to the COVID-19 pandemic only in non-industrial sectors (see Table 12). Therefore, we find some evidence highlighting the importance of the industry type in the relationship between family involvement and financial performance during the pandemic.

**Table 11:** Post-hoc analysis: variations across industries—Industrial sector

Dependent variable: financial performance	Model (1)	Model (2)	Model (3)	Model (4)
COVID-19 growth	− 0.0215	− 0.0585	− 0.0585	− 0.0585
	(0.2452)	(0.0094)	(0.0094)	(0.0094)
Firm age	0.2102	0.2117	0.2118	0.2125
	(0.0106)	(0.0102)	(0.0100)	(0.0098)
Policy	0.0073	0.0072	0.0072	0.0071
	(0.1469)	(0.1542)	(0.1546)	(0.1570)
Family firms x COVID-19 growth		0.1227		
		(0.0027)		
Family-owned firms x COVID-19 growth			0.0365	0.0365
			(0.4867)	(0.4867)
Family-managed firms x COVID-19 growth			0.0783	
			(0.2273)	
Family-managed-owned firms x COVID-19 growth			0.2149	0.2149
			(0.0006)	(0.0006)
Descendant CEO x COVID-19 growth				0.1281
				(0.1060)
Founder CEO x COVID-19 growth				− 0.0594
				(0.5467)
Firm FE Industry FE Country FE	Yes Yes Yes	Yes Yes Yes	Yes Yes Yes	Yes Yes Yes
Constant	− 0.8610	− 0.8670	− 0.8672	− 0.8704
	(0.0100)	(0.0096)	(0.0094)	(0.0092)
Observations	726,878	726,878	726,878	726,878

Notes: This table presents the coefficients and *p*-values (in parentheses) using the FE regressions with robust standard errors clustered at the firm level

**Table 12:** Post-hoc analysis: variations across industries—Non-industrial sectors

Dependent variable: financial performance	Model (1)	Model (2)	Model (3)	Model (4)
COVID-19 growth	− 0.0298	− 0.0582	− 0.0582	− 0.0582
	(0.0461)	(0.0029)	(0.0029)	(0.0029)
Firm age	0.1718	0.1731	0.1727	0.1728
	(0.0002)	(0.0002)	(0.0002)	(0.0002)
Policy	0.0073	0.0072	0.0072	0.0072
	(0.0474)	(0.0516)	(0.0508)	(0.0510)
Family firms x COVID-19 growth		0.0795		
		(0.0083)		
Family-owned firms x COVID-19 growth			0.0331	0.0331
			(0.4539)	(0.4539)
Family-managed firms x COVID-19 growth			0.0881	
			(0.0749)	
Family-managed-owned firms x COVID-19 growth			0.1145	0.1145
			(0.0056)	(0.0056)
Descendant CEO x COVID-19 growth				0.0726
				(0.2184)
Founder CEO x COVID-19 growth				0.1233
				(0.1525)
Firm FE Industry FE Country FE	Yes Yes Yes	Yes Yes Yes	Yes Yes Yes	Yes Yes Yes
Constant	− 0.6556	− 0.6604	− 0.6592	− 0.6594
	(0.0002)	(0.0002)	(0.0002)	(0.0002)
Observations	1,870,656	1,870,656	1,870,656	1,870,656

Notes: This table presents the coefficients and *p*-values (in parentheses) using the FE regressions with robust standard errors clustered at the firm level. Non-industrial sectors are the following: basic materials (ICB 1), consumer goods (ICB 3), consumer services (ICB 5), financials (ICB 8), healthcare (ICB 4), oil and gas (ICB 0), technology (ICB 9), telecommunications (ICB 6), and utilities (ICB 7)

Then, we explored the role of industry concentration using the Herfindahl–Hirschman index (estimated as the sum of squared market shares measured as segment sales at the industry level) (Nawrocki & Carter, 2010). We expect that family firms operating in industries with low concentration (i.e., high degree of competition between firms) are able to adopt more resilient business practices that allow them to decrease costs and financial losses during the pandemic. On the other hand, family firms in highly concentrated industries (i.e., dominated by a few players) would be reluctant to do so, given their large stakes in these sectors and more conservative business practices. Tables 13 and 14 provide empirical support to the above-mentioned prediction. Specifically, as compared to nonfamily firms, family-managed firms and family-owned and managed have better weathered the pandemic only in industries with low concentration, particularly the cluster of founder-led family firms. A possible explanation of this finding is that family firms, who have lower cost of debt (Anderson & Reeb, 2003) and higher employee productivity (Sraer & Thesmar, 2007) than nonfamily firms, were better able to capitalize these two strengths throughout the pandemic in more competitive industries.

**Table 13:** Post-hoc analysis: variations across industries—Highly concentrated industries

Dependent variable: financial performance	Model (1)	Model (2)	Model (3)	Model (4)
COVID-19 growth	0.0436	0.0199	0.0199	0.0199
	(0.0110)	(0.3898)	(0.3899)	(0.3898)
Firm age	0.2174	0.2182	0.2186	0.2181
	(0.0117)	(0.0114)	(0.0113)	(0.0115)
Policy	− 0.0061	− 0.0061	− 0.0062	− 0.0061
	(0.3713)	(0.3660)	(0.3654)	(0.3677)
Family firms x COVID-19 growth		0.0615		
		(0.0734)		
Family-owned firms x COVID-19 growth			0.0810	0.0810
			(0.0818)	(0.0818)
Family-managed firms x COVID-19 growth			0.0284	
			(0.6190)	
Family-managed-owned firms x COVID-19 growth			0.0636	0.0636
			(0.1740)	(0.1741)
Descendant CEO x COVID-19 growth				0.0608
				(0.4055)
Founder CEO x COVID-19 growth				− 0.0277
				(0.6678)
Firm FE Industry FE Country FE	Yes Yes Yes	Yes Yes Yes	Yes Yes Yes	Yes Yes Yes
Constant	− 0.8146	− 0.8177	− 0.8191	− 0.8175
	(0.0129)	(0.0126)	(0.0125)	(0.0127)
Observations	611,592	611,592	611,592	611,592

Notes: This table presents the coefficients and *p*-values (in parentheses) using the FE regressions with robust standard errors clustered at the firm level

**Table 14:** Post-hoc analysis: variations across industries—Low concentrated industries

Dependent variable: financial performance	Model (1)	Model (2)	Model (3)	Model (4)
COVID-19 growth	− 0.1452	− 0.1681	− 0.1680	− 0.1680
	(0.0000)	(0.0000)	(0.0000)	(0.0000)
Firm age	0.1808	0.1812	0.1803	0.1793
	(0.0013)	(0.0013)	(0.0014)	(0.0014)
Policy	0.0095	0.0094	0.0095	0.0095
	(0.0318)	(0.0326)	(0.0317)	(0.0304)
Family firms x COVID-19 growth		0.0729		
		(0.1711)		
Family-owned firms x COVID-19 growth			− 0.1276	− 0.1276
			(0.1048)	(0.1048)
Family-managed firms x COVID-19 growth			0.1641	
			(0.0708)	
Family-managed-owned firms x COVID-19 growth			0.1729	0.1729
			(0.0237)	(0.0237)
Descendant CEO x COVID-19 growth				0.0889
				(0.3223)
Founder CEO x COVID-19 growth				0.4647
				(0.0540)
Firm FE Industry FE Country FE	Yes Yes Yes	Yes Yes Yes	Yes Yes Yes	Yes Yes Yes
Constant	− 0.6939	− 0.6955	− 0.6920	− 0.6884
	(0.0010)	(0.0010)	(0.0010)	(0.0011)
Observations	1,259,064	1,259,064	1,259,064	1,259,064

Notes: This table presents the coefficients and *p*-values (in parentheses) using the FE regressions with robust standard errors clustered at the firm level

Regarding the cross-country differences in the level of economic and institutional development across countries in our sample, we expect that family firms from Anglo-Saxon and European countries were more financially robust to withstand the pandemic due to the better allocation of capital and labor (Hall & Soskice, 2001; La Porta et al., 2013; La Porta et al., 2000), as compared to family firms operating in other parts of the world. Therefore, in Tables 15, 16, 17, and 18, we re-ran our explanatory model for Anglo-Saxon (Table 15), European (Table 16), Asian (Table 17), and other countries (Table 18), separately. We find that the positive effect of family involvement in the firm on financial performance during the pandemic holds for Asian family firms and family firms from other countries (predominantly emerging countries). Interestingly, only the cluster of family-owned and managed European family firms has demonstrated the superior financial performance during the pandemic, while the financial performance of family firms from Anglo-Saxon countries was not different from that of their nonfamily counterparts. One possible explanation of the superior financial performance of Asian family firms and family firms from other emerging markets during the pandemic is their extraordinary growth rates combined with powerful networks (Bennedsen et al., 2022; Keck, 2020; Miroshnychenko et al., 2021). With regard to the above-average financial performance of European firms with family involvement in ownership and management during the pandemic, we believe that this is largely due to their unique cultural and innovative resources (De Massis et al., 2016, 2018a, 2018b) and ability to extract more value from them (Belitski & Rejeb, 2022; Guenther et al., 2022).

**Table 15:** Post-hoc analysis: variations across countries—Anglo-Saxon countries

Dependent variable: financial performance	Model (1)	Model (2)	Model (3)	Model (4)
COVID-19 growth	− 0.3290	− 0.3121	− 0.3121	− 0.3122
	(0.0000)	(0.0000)	(0.0000)	(0.0000)
Firm age	0.1935	0.1937	0.1935	0.1941
	(0.0005)	(0.0005)	(0.0005)	(0.0004)
Policy	− 0.0031	− 0.0031	− 0.0031	− 0.0031
	(0.4609)	(0.4588)	(0.4603)	(0.4557)
Family firms x COVID-19 growth		− 0.0823		
		(0.1867)		
Family-owned firms x COVID-19 growth			− 0.1262	− 0.1262
			(0.1837)	(0.1837)
Family-managed firms x COVID-19 growth			− 0.0386	
			(0.7447)	
Family-managed-owned firms x COVID-19 growth			− 0.0581	− 0.0581
			(0.5340)	(0.5340)
Descendant CEO x COVID-19 growth				0.0547
				(0.6349)
Founder CEO x COVID-19 growth				− 0.4760
				(0.2060)
Firm FE Industry FE Country FE	Yes Yes Yes	Yes Yes Yes	Yes Yes Yes	Yes Yes Yes
Constant	− 0.7382	− 0.7388	− 0.7380	− 0.7404
	(0.0004)	(0.0004)	(0.0004)	(0.0004)
Observations	1,223,829	1,223,829	1,223,829	1,223,829

Notes: This table presents the coefficients and *p*-values (in parentheses) using the FE regressions with robust standard errors clustered at the firm level. Anglo-Saxon countries include Australia, Canada, the UK, and the USA

**Table 16:** Post-hoc analysis: variations across countries—European countries

Dependent variable: financial performance	Model (1)	Model (2)	Model (3)	Model (4)
COVID-19 growth	0.1245	0.1122	0.1123	0.1123
	(0.0000)	(0.0000)	(0.0000)	(0.0000)
Firm age	0.2436	0.2430	0.2427	0.2428
	(0.0007)	(0.0007)	(0.0007)	(0.0007)
Policy	0.0152	0.0152	0.0153	0.0153
	(0.0033)	(0.0032)	(0.0031)	(0.0031)
Family firms x COVID-19 growth		0.0316		
		(0.2335)		
Family-owned firms x COVID-19 growth			0.0034	0.0034
			(0.9240)	(0.9240)
Family-managed firms x COVID-19 growth			0.0012	
			(0.9793)	
Family-managed-owned firms x COVID-19 growth			0.0696	0.0696
			(0.0652)	(0.0652)
Descendant CEO x COVID-19 growth				0.0163
				(0.7650)
Founder CEO x COVID-19 growth				-0.0264
				(0.7161)
Firm FE Industry FE Country FE	Yes Yes Yes	Yes Yes Yes	Yes Yes Yes	Yes Yes Yes
Constant	− 0.9521	− 0.9500	− 0.9486	− 0.9489
	(0.0006)	(0.0006)	(0.0006)	(0.0006)
Observations	978,700	978,700	978,700	978,700

Notes: This table presents the coefficients and *p*-values (in parentheses) using the FE regressions with robust standard errors clustered at the firm level. European countries include Austria, Belgium, Denmark, Finland, France, Germany, Greece, Hungary, Italy, the Netherlands, Norway, Poland, Portugal, Spain, and Sweden

**Table 17:** Post-hoc analysis: variations across countries—Asian countries

Dependent variable: financial performance	Model (1)	Model (2)	Model (3)	Model (4)
COVID-19 growth	− 0.1542	− 0.2332	− 0.2331	− 0.2331
	(0.0001)	(0.0000)	(0.0000)	(0.0000)
Firm age	0.4859	0.4893	0.4864	0.4868
	(0.0000)	(0.0000)	(0.0000)	(0.0000)
Policy	0.0071	0.0069	0.0069	0.0069
	(0.3219)	(0.3352)	(0.3293)	(0.3308)
Family firms x COVID-19 growth		0.2162		
		(0.0080)		
Family-owned firms x COVID-19 growth			0.1748	0.1748
			(0.0272)	(0.0272)
Family-managed firms x COVID-19 growth			0.4355	
			(0.0029)	
Family-managed-owned firms x COVID-19 growth			0.0158	0.0158
			(0.8965)	(0.8965)
Descendant CEO x COVID-19 growth				0.3840
				(0.0055)
Founder CEO x COVID-19 growth				0.6493
				(0.1374)
Firm FE Industry FE Country FE	Yes Yes Yes	Yes Yes Yes	Yes Yes Yes	Yes Yes Yes
Constant	− 1.9830	− 1.9964	− 1.9846	− 1.9860
	(0.0000)	(0.0000)	(0.0000)	(0.0000)
Observations	305,743	305,743	305,743	305,743

Notes: This table presents the coefficients and *p*-values (in parentheses) using the FE regressions with robust standard errors clustered at the firm level. Asian countries include China, Indonesia, Japan, Malaysia, Philippines, Singapore, South Korea, Taiwan, and Thailand

**Table 18:** Post-hoc analysis: variations across countries—Other countries

Dependent variable: financial performance	Model (1)	Model (2)	Model (3)	Model (4)
COVID-19 growth	− 0.5751	− 0.7130	− 0.7130	− 0.7130
	(0.0000)	(0.0000)	(0.0000)	(0.0000)
Firm age	0.1528	0.1736	0.1717	0.1717
	(0.6853)	(0.6432)	(0.6463)	(0.6463)
Policy	− 0.0649	− 0.0669	− 0.0669	− 0.0669
	(0.0381)	(0.0330)	(0.0332)	(0.0332)
Family firms x COVID-19 growth		0.3535		
		(0.0155)		
Family-owned firms x COVID-19 growth			0.1692	0.1692
			(0.3585)	(0.3585)
Family-managed firms x COVID-19 growth			0.4568	
			(0.0276)	
Family-managed-owned firms x COVID-19 growth			0.5045	0.5045
			(0.0340)	(0.0340)
Descendant CEO x COVID-19 growth				0.4568
				(0.0276)
Founder CEO x COVID-19 growth				0.0000
				(.)
Firm FE Industry FE Country FE	Yes Yes Yes	Yes Yes Yes	Yes Yes Yes	Yes Yes Yes
Constant	− 0.4750	− 0.5542	− 0.5471	− 0.5471
	(0.7434)	(0.7008)	(0.7040)	(0.7040)
Observations	89,262	89,262	89,262	89,262

Notes: This table presents the coefficients and *p*-values (in parentheses) using the FE regressions with robust standard errors clustered at the firm level. The cluster of founder CEO firms is not available in this subsample. Other countries include Brazil, Israel, Mexico, South Africa, and Turkey

## Discussion

The global COVID-19 pandemic has fundamentally challenged the way companies operate in today’s world (Belitski, et al., 2022; De Massis & Rondi, 2020; Kraus et al., 2020). In this study, we have examined the important topic of family firms’ superior ability to muddle through adversities (Conz et al., 2020), analyzing the impact of the COVID-19 pandemic on the financial performance of family and nonfamily firms worldwide.

We find that, on average, the financial performance of family firms has been higher during the pandemic as that of nonfamily firms. This finding provides support to the long-term orientation thesis for many family-controlled corporations worldwide. Brigham et al. (2014), De Massis et al. (2012), Lumpkin et al. (2010), Miller & Le Breton-Miller (2005), among many others, have argued that family firms are concerned with the long-term future of the business to support the careers and the financial prosperity of current and later generations. Consequently, many family firms have responded to and recovered better from the shocks caused by the COVID-19 pandemic than their nonfamily counterparts. Indeed, a long-term orientation can allow absorbing environmental shocks.

We also show that the positive family business effect varies across different types of family firms, showing that family-managed and family-owned-managed firms are more resilient than nonfamily and family-owned family firms, while the financial performance of founder-led family firms has been every bit equal to that of descendant-led family firms during the pandemic. In so doing, our work unpacks different degrees of family business resilience to muddle through adversities. We also find that the superior financial performance during the pandemic was largely driven by smaller and younger family firms from non-industrial sectors with low concentration from non-Anglo-Saxon countries. In this context, our work is among the first quantitative studies in this research journey and should be complemented by future work on family business resilience as the research field matures, explicitly accounting for various moderating contingencies potentially affecting the family influence-performance relationship in adverse conditions. Our results remain robust to correcting for endogeneity of family involvement in ownership and/or management, accounting for potential survivorship bias, and to alternative variable definitions and estimation techniques.

### Theoretical implications

Our study has several important theoretical implications for family business and crisis management literatures. First, our findings challenge the stagnation view of family business (Alio, 2004; Bertrand & Schoar, 2006; Neckebrouck et al., 2018), demonstrating with a rich body of evidence from around the world, that family firms, on average, have been more resilient to the COVID-19 pandemic, particularly firms with strong family involvement in management or in both ownership and management. Therefore, the stagnation perspective of family business is thrown into question (Alio, 2004; Bertrand & Schoar, 2006; Neckebrouck et al., 2018), at least regarding the ability to respond to and recover from the adversities caused by the global health crisis via superior financial performance. We hope that our work will spur others to explore in detail potential differences in performance outcomes of family and nonfamily firms during the pandemic, both conceptually and empirically.

Second, our study contributes to the growing literature on crisis management in the context of family firms (De Massis & Rondi, 2020; Czakon et al., 2022; Firfiray & Gomez-Mejia, 2021; Salvato et al., 2020). We provide some preliminary insights on the role of firm-, industry-, and country-level contingencies of the financial performance of family business during the COVID-19 pandemic. In so doing, we answer the research calls of Conz et al. (2023), De Massis & Rondi (2020), and Kraus et al. (2020), among others, to fundamentally advance our understanding of the heterogeneity of family business behavior in times of the pandemic. Moreover, understanding how family influence and moderating contingencies combine to influence financial resilience during the COVID-19 pandemic would seem important to scholars seeking to reveal the drivers behind organizational resilience. We hope that this investigation can serve as a springboard for future studies examining the resilience of firms with different ownership and management structure in times of environmental shocks.

In responding to the call of Conz et al. (2020) to empirically analyze family business resilience in longitudinal, cross-industry, and cross-country settings, we identify variations across family-owned, family-managed, and family-owned and managed, founder-led and descendants-led family firms thereby also addressing the call of Memili & Dibrell (2019) to pay more attention to family firm heterogeneity in examining performance outcomes. In so doing, our study challenges the literature highlighting the extraordinary performance of founder CEOs in family business (Miller et al., 2007a; Wagner et al., 2015), identifying the conditions under which descendant-led family firms also create financial value for family firms.

### Practical implications

Our study also has important practical implications. Owners, managers, and advisors of family and nonfamily firms are increasingly required to help their firms develop organizational resilience to be ready for future crises (DesJardine et al., 2019; Forbes, 2020; Ortiz-de-Mandojana & Bansal, 2016) and black swan events (Taleb, 2007; Orlik, & Veldkamp, 2014). Our results show that resilience to environmental shocks is particularly pronounced in family firms compared to their nonfamily counterparts. Thus, nonfamily business owners, managers, and advisors should consider strengthening the resilience pillar of their corporate strategy to keep up with competitors and be sustainable over time. Investors too must pay attention to a firm’s ownership and management structure in evaluating the potential resilience of the business to future crises. At the same time, our results warrant caution in relying only on founders in the top management team of family firms, as they might be as useful in weathering adverse environmental events as other family members.

Our results also contribute to the more general debate on the economic and financial implications of the COVID-19 pandemic for the global economy (Al-Awadhi et al., 2020; Kurowski et al., 2020; OECD, 2020). Specifically, we empirically show with a rich body of evidence collected from around the world that family firms are more resilient than their nonfamily counterparts when facing environmental shocks. We also identify variations across different types of family firms, industries, and countries. Our results suggest that the entrepreneurial organization type and its governance must be considered when establishing national financial assistance policies, particularly for firms with different ownership and management structure. Such policies can be used to promote and increase the adoption of practices to foster resilience among nonfamily firms and family-owned firms.

### Limitations and future research

We also acknowledge some limitations of our study that open up several avenues for future research. As most studies on the long-term orientation of family business (De Massis et al., 2012; Gentry et al., 2016; Le Breton-Miller & Miller, 2006; Miller & Le Breton-Miller, 2005), we do not directly measure different components of long-term orientation. Nonetheless, we use a wide range of firm-, industry-, and country-level controls, along with different specification models in our study to rule out possible alternative explanations of our principal findings. Therefore, we encourage future research studying the resilience of family vs. nonfamily businesses in times of adverse environmental events to assess the validity of our principal findings by explicitly measuring family business futurity, continuity, and perseverance. In addition, it would be interesting to understand the role of individual traits of family members (i.e., motivation, education, experience) involved in ownership and/or management in the resilience of family firms.

The secondary data sources we used, and all their inherent limitations, call for future research using primary data or the combination of primary and secondary data to analyze the financial performance of family firms vs. nonfamily firms during the pandemic. This would allow further assessing the generalizability of our findings. We also encourage scholars to study the resilience of family business behavior using qualitative (De Massis & Kammerlander, 2020; De Massis & Kotlar, 2014; Van Burg et al., 2022) and mixed research methods (Molina-Azorin, 2010; Reilly & Jones, 2017) to disentangle the effects of cultural differences and intraorganizational dynamics on family business resilience. In addition, these methods can also allow to explore the link between family business and COVID-19 through the lens of crisis-as-process concept (Williams et al., 2017).

In this work, we have only analyzed publicly traded firms. Thus, we encourage future studies to look at the resilience of private family firms with idiosyncratic investment strategies (Bartz & Winkler, 2016; Gomez-Mejia et al., 2014; Santoro et al., 2021) and not affected by financial market pressures (Carney et al., 2015; Huybrechts et al., 2012). We also hope that others will examine the non-financial performance of family business during the pandemic, given the importance of non-financial goals in family firms compared to their nonfamily counterparts (Chua et al., 2018; Kotlar & De Massis, 2013).

## Conclusion

Our study examined the impact of the COVID-19 pandemic on the financial performance of family and nonfamily firms worldwide. Consistent with the long-term view of family business, we find that family firms on average had higher financial performance during the pandemic compared to their nonfamily counterparts, and this resilience effect is particularly strong for firms with active involvement of the controlling family either in management or in both ownership and management. We also show that this positive family business effect varies substantially across different family firms, industrial sectors, and countries. Taken together, our study highlights that family involvement in the firm can lead to robust financial performance in the wake of adverse environmental events, but the magnitude of this impact is largely conditional on the type of family involvement, firm type, industry, and country.

## Data Availability

Our paper relies upon licensed data coming from Thomson Reuters Eikon and NRG Metrics. Given the proprietary nature of the afore-mentioned data sources, we provide all the detailed information about the construction of our sample in the Data section of the paper. Moreover, the data dictionary that contains a description of all variables used in the paper is provided in the paper along with all the summary statistics for all the variables used.

## Footnotes

1 Our study sample covers the following countries: Australia, Austria, Belgium, Brazil, Canada, China, Denmark, Finland, France, Germany, Greece, Hungary, Indonesia, Israel, Italy, Japan, Malaysia, Mexico, the Netherlands, Norway, Philippines, Poland, Portugal, Singapore, South Africa, South Korea, Spain, Sweden, Taiwan, Thailand, Turkey, the UK, and the USA.

2 China is represented in our sample by listed firms from Hong Kong due to the poor quality of financial reporting in the rest of the country (Wang & Wu, 2011).
